# Capability and dependency in the Newcastle 85+ cohort study. Projections of future care needs

**DOI:** 10.1186/1471-2318-11-21

**Published:** 2011-05-04

**Authors:** Carol Jagger, Joanna C Collerton, Karen Davies, Andrew Kingston, Louise A Robinson, Martin P Eccles, Thomas von Zglinicki, Carmen Martin-Ruiz, Oliver FW James, Tom BL Kirkwood, John Bond

**Affiliations:** 1 Institute for Ageing and Health, Newcastle University, Campus for Ageing and Vitality, Newcastle upon Tyne NE4 5PL UK; 2 Institute of Health and Society, Newcastle University, Baddiley-Clarke Building, Richardson Road, Newcastle upon Tyne NE2 4AX UK

## Abstract

**Background:**

Little is known of the capabilities of the oldest old, the fastest growing age group in the population. We aimed to estimate capability and dependency in a cohort of 85 year olds and to project future demand for care.

**Methods:**

Structured interviews at age 85 with 841 people born in 1921 and living in Newcastle and North Tyneside, UK who were permanently registered with participating general practices. Measures of capability included were self-reported activities of daily living (ADL), timed up and go test (TUG), standardised mini-mental state examination (SMMSE), and assessment of urinary continence in order to classify interval-need dependency. To project future demand for care the proportion needing 24-hour care was applied to the 2008 England and Wales population projections of those aged 80 years and over by gender.

**Results:**

Of participants, 62% (522/841) were women, 77% (651/841) lived in standard housing, 13% (106/841) in sheltered housing and 10% (84/841) in a care home. Overall, 20% (165/841) reported no difficulty with any of the ADLs. Men were more capable in performing ADLs and more independent than women. TUG validated self-reported ADLs. When classified by 'interval of need' 41% (332/810) were independent, 39% (317/810) required help less often than daily, 12% (94/810) required help at regular times of the day and 8% (67/810) required 24-hour care. Of care-home residents, 94% (77/82) required daily help or 24-hour care. Future need for 24-hour care for people aged 80 years or over in England and Wales is projected to increase by 82% from 2010 to 2030 with a demand for 630,000 care-home places by 2030.

**Conclusions:**

This analysis highlights the diversity of capability and levels of dependency in this cohort. A remarkably high proportion remain independent, particularly men. However a significant proportion of this population require 24-hour care at home or in care homes. Projections for the next 20 years suggest substantial increases in the number requiring 24-hour care due to population ageing and a proportionate increase in demand for care-home places unless innovative health and social care interventions are found.

## Background

People aged 85 years or over (the so-called oldest-old) constitute the fastest growing age-group within the population [1]. A concern is that increasing life extension will be accompanied by higher levels of disease which translate into disability, dependency and increasing demands for health and social care services. Severity of disability as measured by restrictions in self-reported Activities of Daily Living (ADLs), likelihood of cognitive impairment, and of being dependent on formal and informal support all increase with age [2]. Nowadays the highest proportion of dependent older people are aged 80 or over [2]. However, increasing longevity does not necessarily result in very high levels of disability and dependency because of the high levels of mortality among the most disabled or dependent at any given time [3].

In Europe and North America a handful of studies have begun to document the changing disability and dependency profiles of the oldest old [4-11]. Such profiles have been used in planning health and social care provision and the projection of future needs for services [12-14].

In the UK and many other European countries there is universal free health care for dependent older people [15]. Long-term care has long been provided at home by families and informal carers while the proportion of people aged 65 or over resident in long-term care institutions has remained at around 5% for the last 50 years despite demographic changes [15]. Recent years have shown a diversification of different types of assisted living facilities such as sheltered housing in the UK where older people live in specially adapted housing but are supported by home-based services and receive regular visits from a warden [15]. For ageing populations world wide a key challenge will be the projected decline in the traditional sources of formal and informal carers to support people living at home and in long-term residential or nursing home care [16].

This paper complements existing investigations of the oldest old and uses baseline data from the Newcastle 85+ study [17] to describe the capability and dependency of 85 year olds living in north east England and to predict the future need for formal and informal support in England and Wales.

## Methods

The target population for the Newcastle 85+ Study was all surviving adults born in 1921, who turned 85 in 2006 when the study commenced, and permanently registered with a participating general practice in Newcastle or North Tyneside NHS Primary Care Trusts in north east England. Full details of the design of the study and recruitment of participants have been reported [17-19]. The study was approved by the Newcastle and North Tyneside 1 research ethics committee (Ref: 06/Q0905/2).

Participation entailed a detailed multidimensional health assessment and review of general practice medical records; participants could decline elements of the protocol. Trained research nurses interviewed participants in their usual residence (own home or institution). Written informed consent was obtained from participants. Where people lacked capacity to consent an opinion was sought from a relative or carer (a "consultee") according to the requirements of the UK Mental Capacity Act. A consultee opinion was also obtained if a participant was initially judged to have capacity to consent but subsequently scored below 17 on the Standardised Mini-Mental State Examination (SMMSE) [20]. Proxy informants were used in interviews where cognitive impairment was judged to limit the ability of participants to provide reliable answers. Information collected included: basic socio-demography (gender, type of housing and household composition); cognitive status assessed by the SMMSE; continence status graded on a combination of frequency and severity of urinary symptoms [21]; and self-reported ability to perform 17 Activities of Daily Living (ADLs) (Table 1). Participants, who received help with ADLs were also asked who provided the help and how often help was received. Participants also performed a timed up and go (TUG) test [22]; the time taken to rise from a standard chair, walk 3 metres, turn, walk back and sit down was recorded. Information on diseases was collected from a review of GP records; data was extracted by research nurses. Further details of the interview schedules used in the study are available at: http://www.ncl.ac.uk/iah/research/programmes/85plus.htm

**Table 1:** Self-reported activities of daily living^§^

**Basic activities of daily living**:
feeding self - including cutting up of food
washing face and hands
washing all over*
getting in and out of bed
getting on and off the toilet*
getting in and out of a chair
dressing and undressing*
cutting own toenails

**Instrumental activities of daily living:**

light housework
heavy housework*
preparing and cooking a hot meal*
shopping for groceries*
taking medication*
managing money*

**Mobility items:**

getting around the house
going up and down stairs/steps
walking at least 400 yards

**Response categories:**

can do on own without difficulty
can do on own but with difficulty
can do on own but only with aid or appliance
unable to do without personal help

^§ ^15 of the 17 activities assessed were taken from the 18 activities in the Groningen Activity Restriction Scale (GARS) [36]. The 2 additional items (medicines and money) came from the Leiden 85+ study [4] (which used the GARS and added these items). The phrasing of activities and response options were altered from GARS and made similar to the MRC Health Services Research Collaboration questionnaire [37].

* Activities for which participants who received help were asked who provided the help, how often help was received and whether the help received met their needs.

Capability of participants was based on ability to undertake ADLs. Dependency was estimated using Isaacs' and Neville's concept of 'interval of need', which classifies people on the basis of their cognitive status, continence status and the lapsed time between periods when participants require help with ADLs [23]. Four categories were used: independent (participants for whom supervision or help for any activity was not essential); long-interval dependency (required help less often than daily); short-interval dependency (required help at regular intervals each day) and critical-interval dependency (required 24-hour care since help required potentially at any time or participant required constant supervision). Participants were allocated to categories in the following way: critical interval - having a SMMSE score of less than 10, or having severe or profound urinary incontinence with inability to dress or undress without help, or unable to perform, without help, any of: getting on and off the toilet, or getting in and out of a chair, or feeding oneself; short interval - unable to perform, without help, any of: getting in and out of bed, dressing and undressing, preparing and cooking a hot meal, taking medication or washing face and hands; long interval - unable to perform, without help, any of: washing all over, shopping for groceries, light housework, heavy housework, managing money or cutting own toenails. The remainder were defined as independent. Participants were classified as missing for this variable only if they could not be unequivocally categorised.

Health and social care service use was ascertained by self-report and from information extracted from general practice records by the research nurses (Table 2).

**Table 2:** Health and social care services

Self-report: *In previous four weeks, any contact with*:
• community nurse (including district nurse, practice nurse, private nurse, MacMillan nurse, Marie Curie nurse and other specialist community nurses)
• chiropodist (NHS or private)
• physiotherapist
• occupational therapist
• speech therapist
• dietician
• home-care service (social services, voluntary agency or private)
• meals service
• day sitter
• night attendant
• social worker

*In previous three months:*

• any outpatient attendance (with number of attendances)
• any 'accident and emergency' attendance (with number of attendances)
• any use of emergency ambulance

*In previous 12 months:*

• any overnight hospital admission (with total length of stay)
• any respite care (care home or hospital, with total length of stay)
• any 'day hospital' attendance
• any use of other intermediate care services

**General practice records**

*In previous 12 months:*

• any consultation with own general practitioner, including 'out of hours' contacts with own general practitioner (with number of consultations)
• general practice 'out of hours' service (with number of consultations)
• practice nurse (excluding district nurse) (with number of consultations)

### Statistical methods

As the TUG test is a more objective measure of physical function we validated participants' self-report of ability in mobility items (getting around the house, getting in and out of a chair, shopping, going up and down stairs, walking at least 400 yards) by comparing the median time to completion and proportion unable to complete the TUG test by response category for each mobility item and separately by gender. Individual responses to ADL items were compared between genders by Mann-Whitney (M-W) tests. Socio-demographic inequalities in interval-need dependency were examined using Kruskall-Wallis (K-W) tests (P values were two sided) and polytomous regression models with adjustment for gender and number of chronic diseases (disease count [17]), the latter being a proxy for disease burden. Polytomous regression models with adjustment for gender were also used to explore the association between interval-need dependency and health and social care service use with significance assessed by the Wald Chi-squared (Wald χ^2^). In general we excluded missing values from the analysis and calculated percentages from the number of valid responses. Where individual items were missing within the SMMSE we compared scoring the missing item as zero or the maximum possible for that item with data retained only if a participant was classified in the same category (<10, 10+) in either case. We used version 1.2 of the data set.

To assess the potential future dependency needs of an ageing population we assumed that 85 year olds, and their dependency levels, were an estimate for the national (England and Wales) population aged 80 years and over. This assumption was reasonable for two reasons. Firstly 85 is the average age of the 80 years and over age group as the median age of this age group from the population projections varied from 84.9 years (men: 84.3 years, women: 85.2 years) in 2010 to 85.4 years (men: 85.1 years, women: 85.6 years) in 2030 [24]. Secondly the prevalence of dependency in very old cohorts appears to be relatively constant as they age further due to the increased mortality in the most dependent[25]. Therefore we applied the proportions in the interval-need-dependency groups by gender for our population of 85 year olds to the 2008-based population projections through to 2030 for men and women separately [24].

## Results

**Figure 1:**
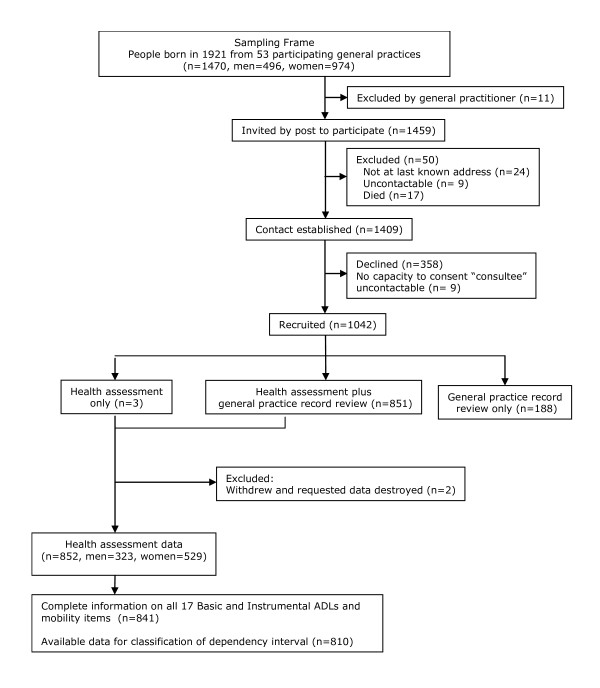
**Recruitment profile for the Newcastle 85+ Study**.

In total 1042 participants were recruited to the Newcastle 85+ study (71.7% of those eligible and alive); health assessment data were available on 852 participants of whom 841 (98.7%, 319 men, 522 women) had complete information on all 17 ADLs and formed the study population for analysis (Figure 1). As reported elsewhere, participants were broadly representative of people of this age in England and Wales in terms of gender, being resident in care homes and living alone [17].

### Sociodemographics

Of the 841 participants for whom complete information on ADLs were available, 77% (651/841) lived in standard housing, 13% (106/841) in sheltered accommodation and 10% (84/841) in a care home (data not shown).

### Self-reported capability to perform activities of daily living

Participants alone provided self-report of capability in daily activities in 84% (706/841) of cases with proxy informants also contributing in a further 12% (105/841) and for 4% (30/841) proxy informants alone provided information. Over 90% of men and women could feed themselves (792/841) and wash their face and hands (789/841) without difficulty whilst cutting toenails was the activity with which most men and women had difficulty; only 31% (160/522) of women and 41% (131/319) of men could do this without difficulty (Table 3). Women were significantly more likely than men to be limited in 13 of the 17 ADLs (feeding self, getting in and out of bed, getting on and off the toilet, taking medication, getting around in the house, preparing and cooking hot meal, managing money, washing all over, doing heavy housework, shopping for groceries, going up and down stairs, walking at least 400 yards, and cutting own toenails). Self-report of ability to perform the activities concerned predominantly with lower limb mobility was strongly related to the ability to complete and the time taken to complete the timed up and go test (Table 4) thus validating the self-reports.

**Table 3:** Capability and restriction in activities of daily living, by sex.

	No difficulty doing alone		Some difficulty doing alone		Can only do with an aid		Unable to do alone, need help		
	**Men** **%** **(N = 319)**	**Women % (N = 522)**	**Men** **% (N = 319)**	**Women % (N = 522)**	**Men** **% (N = 319)**	**Women % (N = 522)**	**Men** **% (N = 319)**	**Women % (N = 522)**	P value*

Feeding self including cutting up food	96.9	92.5	1.9	3.1	0.3	0.2	0.9	4.2	0.009

Washing face and hands	95.6	92.7	1.9	2.9	0.0	0.0	2.5	4.4	0.09

Getting in and out of bed	88.1	81.8	6.0	8.4	1.9	3.6	4.1	6.1	0.015

Getting on and off toilet	85.6	79.3	4.4	5.9	5.6	8.8	4.4	5.9	0.023

Taking medication	85.6	79.9	2.8	4.4	0.3	1.3	11.3	14.4	0.044

Dressing and undressing	84.3	81.0	8.2	8.6	0.0	0.4	7.5	10.0	0.21

Light housework	83.1	78.2	4.4	5.7	0.3	1.3	12.2	14.8	0.1

Getting around the house	82.4	74.3	6.3	5.7	7.8	14.4	3.4	5.6	0.004

Preparing and cooking a hot meal	81.8	74.7	5.0	5.7	0.3	0.4	12.9	19.2	0.014

Managing money	80.3	72.8	6.0	6.3	0.0	0.2	13.8	20.7	0.011

Getting in and out of a chair	79.6	77.8	15.4	14.6	0.9	2.5	4.1	5.2	0.45

Washing all over	74.6	61.1	8.8	9.6	5.0	10.5	11.6	18.8	<0.0001

Heavy housework	69.9	43.3	10.7	14.6	0.3	0.2	19.1	42.0	<0.0001

Shopping for groceries	61.8	36.8	9.1	11.9	2.8	1.0	26.3	50.4	<0.0001

Going up and down stairs/steps	61.1	45.6	21.0	25.5	6.0	8.2	11.9	20.7	<0.0001

Walking at least 400 yards	60.5	46.9	13.2	11.3	15.0	18.0	11.3	23.8	<0.0001

Cutting own toenails	41.1	30.7	23.8	15.5	0.3	0.0	34.8	53.8	<0.0001

Values are percentages.

*Mann-Whitney U test for gender difference

**Table 4:** Median time in seconds to complete timed up and go (TUG) test and proportion unable to complete, by mobility item response and gender.

	No difficulty doing alone		Some difficulty doing alone		Can only do with an aid		Unable to do alone need help		
	**Median time (secs)**	% miss-ing*	**Median time (secs)**	% miss-ing*	**Median time (secs)**	% miss-ing*	**Median time (secs)**	% miss-ing*	P value**

**Men**									

Getting around the house	12.2	4.2	26.4	10.0	30.1	16.0	57.4	90.9	<0.0001

Getting in and out of a chair	12.3	3.2	18.1	12.2	25.0	0.0	.	100.0	<0.0001

Shopping for groceries	11.2	2.0	14.6	6.9	24.0	11.1	20.8	23.8	<0.0001

Going up and down stairs/steps	11.3	3.1	14.5	4.5	30.7	5.3	25.6	44.7	<0.0001

Walking at least 400 yards	11.2	2.6	13.6	4.8	20.3	8.3	21.3	44.4	<0.0001

									

**Women**									

Getting around the house	13.9	5.4	28.0	16.7	37.3	25.3	35.0	93.1	<0.0001

Getting in and out of a chair	14.2	6.2	24.8	19.7	50.0	46.2	39.8	96.3	<0.0001

Shopping for groceries	12.3	2.1	14.1	8.1	16.2	0.0	22.0	24.0	<0.0001

Going up and down stairs/steps	12.6	4.6	16.2	6.0	27.2	7.0	37.8	46.3	<0.0001

Walking at least 400 yards	12.4	5.3	16.5	1.7	17.4	9.6	31.0	39.5	<0.0001

*%missing through inability to complete TUG

**Kruskal-Wallis test for difference in TUG between ADL response categories

### Cognitive status

Using an SMMSE cut-point of ≤ 21, 13% (105/836) were moderately or severely cognitively impaired; 3% (25/836) scored < 10.

### Interval-need dependency

Of the 841 participants with complete data on ADLs, 31 (9 men, 22 women) had missing data on continence or SMMSE and therefore could not be classified for interval-need dependency. When classified by interval-need dependency, 41% (332/810) were independent; 39% (317/810) were long-interval dependent requiring help less than daily; 12% (94/810) were short-interval dependent requiring help at regular intervals every day and only 8% (67/810) of the cohort were critical-interval dependent requiring 24-hour care (Table 5). Of those in care homes, 61% (50/82) required 24-hour care (critical-interval) and a further 33% (27/82) required help at regular intervals throughout the day (short-interval) (Table 5). Women required care at significantly shorter intervals than men (M-W, P < 0.0001). Of those classified as critical-interval dependent, 75% (50/67) lived in a care home and for the remainder the main provider of help was a child (47%, 8/17), social services (35%, 6/17) or a spouse (18%, 3/17). For those living at home with short-interval dependency the main provider of help was a spouse (34%, 23/67), a child (31%, 21/67) or social services (22%, 15/67) whilst the majority of help for those living at home with long-interval dependency was provided by a child (37%, 115/313) (Table 5). Non-institutionalised participants who lived with others (but not solely a spouse) required care more frequently (K-W, χ^2 ^= 8.482; P = 0.014) and differences remained after adjustment for gender and number of chronic diseases (Table 6).

**Table 5:** Interval-need dependency, by socio-demographics and main provider of help with daily activities.

	Independent	Long interval	Short interval	Critical interval	Total
**Gender**					
Men	57.7 (179)	26.1 (81)	9.7 (30)	6.5 (20)	38.3 (310)
Women	30.6 (153)	47.2 (236)	12.8 (64)	9.4 (47)	61.7 (500)
All	41.0 (332)	39.1 (317)	11.6 (94)	8.0 (67)	100.0 (810)

**Housing**					
Standard	48.1 (301)	41.5 (260)	8.1 (51)	2.2 (14)	77.3 (626)
Sheltered	29.4 (30)	52.0 (53)	15.7 (16)	2.9 (3)	12.6 (102)
Institution	1.2 (1)	4.9 (4)	32.9 (27)	61.0 (50)	10.1 (82)

**Living arrangements^1^**					
Alone	43.6 (192)	47.0 (207)	7.5 (33)	1.8 (8)	60.6 (440)
With spouse only	53.4 (109)	33.3 (68)	11.8 (24)	1.5 (3)	28.1 (204)
With other(s)	35.4 (29)	45.1 (37)	12.2 (10)	7.3 (6)	11.3 (82)

**Main provider of help with activities^2 ^of daily living^1^**					
					
No-one	81.5 (269)	18.2 (57)	0.0 (0)	0.0 (0)	44.8 (326)
Spouse	4.2 (14)	13.4 (42)	34.3 (23)	17.6 (3)	11.3 (82)
Children	8.8 (29)	36.7 (115)	31.3 (21)	47.1 (8)	23.8 (173)
Other relatives	2.1 (7)	9.6 (30)	4.5 (3)	0.0 (0)	5.5 (40)
Friend/neighbour	0.3 (1)	7.7 (24)	1.5 (1)	0.0 (0)	3.6 (26)
Home care - social services	0.3 (1)	6.4 (20)	22.4 (15)	35.3 (6)	5.8 (42)
Home care - voluntary	0.0 (0)	0.0 (0)	1.5 (1)	0.0 (0)	0.1 (1)
Home care - private	2.7 (9)	8.0 (25)	4.5 (3)	0.0 (0)	5.1 (37)

Values are percentages (numbers).

**Table 6:** Regression models of interval-need dependency by sociodemographics.

	Model 1 (unadjusted)	Model 2 (adjusted for gender)	Model 3 (adjusted for gender and disease)
**Housing**			
Standard	1	1	1
Sheltered	1.32 (1.13 to 1.53)	1.29 (1.11 to 1.50)	1.26 (1.09 to 1.46)
Institution	6.63 (5.61 to 7.83)	6.35 (5.39 to 7.49)	5.37 (4.44 to 6.48)

**Living Arrangements^1^**			
Alone	1	1	1
With spouse only	0.94 (0.83 to 1.06)	1.07 (0.94 to 1.22)	1.05 (0.92 to 1.19)
With other	1.27 (1.07 to 1.51)	1.28 (1.08 to 1.51)	1.18 (1.00 to 1.40)

Odds ratios (95 percent confidence limits).

1. Excludes 82 participants resident in a long-term care institution.

The self-reported frequency of help received validated the interval-need dependency classifications. The majority of participants classified as critical-interval dependent received help several times a day with dressing or undressing (79%, 53/67), taking medication (79%, 53/67) and toileting (70%, 47/67). Those classified as long-interval dependent that required help less than daily did so mostly because they needed help with heavy housework (7.6%, 24/317).

### Health and social care service use

Participants with higher levels of interval-need dependency were more likely to have used health services (Table 7).

**Table 7:** Use of health and social care services, by interval-need dependency group.

	In-dependent	Long interval	Short interval	Critical Interval	Total	P value
**Previous 4 weeks**						
Any contact with community nurse^1^	2.4 (8)	12.7 (40)	21.3 (20)	17.9 (12)	10.0 (80)	*<0.0001*
Chiropody (NHS or private)^1^	10.9 (36)	36.5 (115)	31.9 (30)	43.3 (29)	26.1 (210)	*<0.0001*
Any homecare (social services, voluntary agency or private)^1,2^	5.5 (18)	24.6 (77)	54.6 (36)	70.6 (12)	19.8 (143)	*<0.0001*

**Previous 3 months**						
Any outpatient attendance^1^	30.3 (100)	34.5 (109)	31.2 (29)	26.6 (17)	31.8 (255)	
*Median (inter-quartile range) number of outpatient attendances*^1,3^	*1 (1 to 2)*	*1 (1 to 2)*	*1 (1 to 2)*	*1 (1 to 3)*	*1 (1 to 2)*	
Any 'accident and emergency' attendance^1^	5.1 (17)	6.9 (22)	13.8 (13)	9.1 (6)	7.2 (58)	<0.05
*Median (inter-quartile range) number of 'accident and emergency' attendances*^1,3^	*1 (1 to 1)*	*1 (1 to 1)*	*1 (1 to 2)*	*1 (1 to 1)*	*1 (1 to 1)*	
Any emergency ambulance use^1^	2.7 (9)	5.0 (16)	12.8 (12)	7.5 (5)	5.2 (42)	<0.001

**Previous 12 Months**						
Any overnight hospital admission^1^	14.8 (49)	26.3 (83)	22.1 (21)	38.8 (26)	22.1 (179)	
*Median (inter-quartile range) total stay in hospital (days)^1,3^*	*4 (1 to 12)*	*7 (3 to 16)*	*14 (7 to 41)*	*20 (7 to 47)*	*7 (3 to 19)*	*<0.0001*
Any respite care^1,4^	0.0 (0)	1.3 (4)	9.8 (8)	19.6 (10)	2.8 (22)	
*Median (inter-quartile range) total stay in respite care (days)^1,3^*	*0 (0 to 0)*	*13 (8 to 26)*	*14 (7 to 22)*	*21 (6 to 25)*	*14 (7 to 22)*	*<0.0001*

Any 'Day Hospital' attendance^1^	7.2 (24)	7.0 (22)	8.6 (8)	9.1 (6)	7.4 (60)	
Any other 'Intermediate Care' contact^1^	3.6 (12)	9.6 (30)	10.9 (10)	13.4 (9)	7.6 (61)	*<0.005*
Any consultations with own general practitioner (including 'out of hours' contacts)^5^	94.0 (312)	93.4 (296)	94.7 (89)	92.5 (62)	93.7 (759)	
*Median (inter-quartile range) number of own general practitioner consultations^3,5^*	*5 (2 to 8)*	*5 (3 to 9)*	*5 (2 to 8)*	*6 (4 to 10)*	*5 (2 to 8)*	
Any consultations with 'out of hours' general practice service^5^	3.6 (12)	5.7 (8)	10.6 (10)	13.4 (9)	6.0 (49)	<0.005
Any consultations with practice nurse^5^	88.0 (292)	78.5 (249)	60.6 (57)	35.8 (24)	76.8 (622)	
*Median (inter-quartile range) number of practice nurse consultations^3,5 ^*	*3 (1 to 5)*	*2 (1 to 4)*	*1 (0 to 3)*	*0 (0 to 1)*	*2 (1 to 4)*	*<0.0001*

Values are percentages (numbers) unless otherwise stated.

1 - Data from health assessment.

2 - Community dwelling residents only.

3 - Median (inter-quartile range) based on participants attending/consulting service only.

4 - Excludes participants resident in a long-term care institution for the last 12 months

5 - Data from general practice record review.

#### Health service use

For participants living at home and in care homes, higher levels of interval-need dependency were significantly associated with: hospital admission, use of respite care, use of intermediate care other than day hospital and use of an out of hours doctor service in the previous year; use of an emergency ambulance and attendance at 'accident and emergency', in the previous 3 months; community nurse contact or use of NHS or private chiropody services, in the previous 4 weeks. Participants with higher levels of interval-need dependency were significantly less likely to have consulted a practice nurse in the previous year. When adjusted for the effect of gender, only hospital admission (Odds Ratio [OR] 1.29, 95% Confidence Interval [95%CI] [1.16 to 1.44]), the use of respite care (OR 1.22, 95% CI [1.16 to 1.28]) in previous year, and use of chiropody (OR 1.36, 95% CI [1.22 to 1.52]) in previous 4 weeks suggested a strong association with critical-interval dependency. Only use of respite care (OR 1.20, 95% CI [1.15 to 1.26]) in previous year showed a strong association with short-interval dependency. Furthermore, those in the critically dependent group who received respite care in the 12 months before interview (n = 10) were compared with those who had not received respite care (n = 41) in terms of participant characteristics (gender, marital status, living arrangements) and carer characteristics (relationship to participant). No significant differences were found between the groups for gender, marital status or living arrangements. However more who received respite care had a child as the main carer (87% v 27%, p = 0.02) though these results should be viewed with caution due to small numbers. Less than 4% of participants had had any contact in the previous 4 weeks with a physiotherapist (27/808), occupational therapist (14/808), speech therapist (5/808), or dietician (8/808); use of these services by dependency group was not explored.

#### Social care service use

For participants living at home, higher levels of interval-need dependency were significantly associated with contact with home-care services in the previous 4 weeks, differences remaining after adjustment for gender (OR 1.92, 95% CI [1.61 to 2.29], critical-interval dependency). For participants living at home, only 7% (48/724) reported receiving any meals service in the previous 4 weeks and less than 4% reported any contact in the previous 4 weeks with a night attendant (3/724) or day sitter (8/725). For participants living at home or in an institution, less than 4% (32/804) reported any contact with a social worker in the previous four weeks. Use of these services by dependency group was therefore not explored.

### Future projections

**Figure 2:**
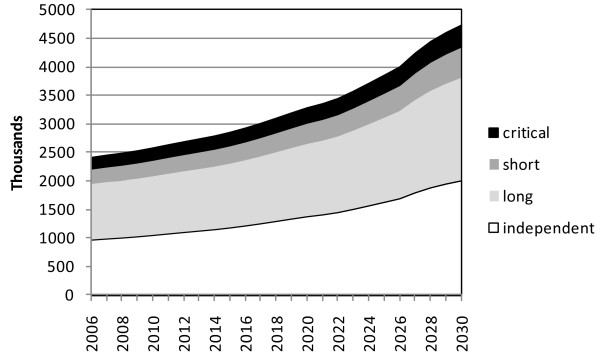
**Projected numbers (thousands) in England and Wales aged 80+ with categories of interval-need dependency**.

Assuming that the proportions of 85 year olds in each interval-need dependency category remain constant and are an estimate for the 80+ population, the numbers in England and Wales with critical-interval dependency, requiring help potentially at any time of the day or night, are projected to rise by 82% between 2010 to 2030, from 216,000 to 392,000 (Figure 2) and those with short-interval dependency by 83% from 303,000 to 553,000. The number of people aged 80 or over who remain independent is projected to rise by 94% from 1,057,000 to 2,047,000. Assuming that the balance between home-based care and institutional care for people with short or critical-interval dependency remains constant then the number of care-home places required in 2030 in England and Wales is projected to be 630,000.

## Discussion

Despite considerable morbidity [17], people aged 85 years born in 1921 living in Newcastle and North Tyneside in the UK are remarkably independent. Using Isaacs' and Neville's [23] concept of interval need we estimate that about two-fifths of this age cohort are independent, two-fifths were long-interval dependent requiring help less often than daily, around one-in-ten were short-interval dependent requiring help at regular times of the day) and under one-in-ten were critical-interval dependent requiring 24-care by formal and informal carers. Men were estimated to be more independent than women (58% compared with 31%). This reflected gender differences in the need for help with activities of daily living (see Table 3). Not surprisingly residents in care homes were more dependent even after adjusting for gender and disease compared with participants living at home or in sheltered accommodation (see Table 6). Of those not resident in care homes, two-fifths of participants received no help with activities of daily living; for two-fifths the reported main helper was a family member, friend or neighbour and in only one fifth was the main helper a formal carer (see Table 5). We estimate that the number of people aged 80 years or over in England and Wales who are independent will increase by 94% from 2010 to 2030. We estimate similar increases in this period for the number of people aged 80 years or over who are long-interval (79%), short-interval (83%) and critical-interval (82%) dependent. Three-quarters of study participants with critical-interval dependency and almost a third of those with short-interval dependency were living in care homes. Assuming constant prevalence of interval-need dependency and that the balance of care homes to other ways of supporting dependent older people also remains constant, these estimates would imply that there will be an 89% increase in the demand for residential and nursing home places over the same period.

The so-called oldest old (generally defined as people aged 85 years or over) have been poorly represented in ageing research. Data about the very old are routinely based on relatively small sample sizes because of the relatively small numbers of people aged 85 years or over sampled from populations of people aged 60 or 65 years and over, which is compounded by attrition and non-response [26-28]. The oldest old represent an extremely heterogeneous age group covering 20 years or more of the observed lifespan making generalisations about this age group particularly problematic. Narrow age-band cohort studies of very old people more readily reflect the true diversity within the population than those of wider age-band cohorts that are confounded by the differential effects of increasing age. The Newcastle 85+ study is one of a small number of studies that have the advantage of a relatively homogeneous and stable study population, of sufficient size and that recruited both participants living at home and in long-term care institutions including people with cognitive impairment. The challenges of researching very old people in terms of the reliability and internal validity of self-reported data [29,30], however, remain. Non-response bias, however, that would normally impact on population estimates of care need, surprisingly, does not appear to reflect institutionalisation and increasing disability and dependency [17]. Caveats about the external validity of estimates based on a single urban area should be highlighted. However, our study population of people born in 1921 shows remarkable similarity to the England and Wales population [17]. This includes ethnicity since projections based on the 2001 national census suggest that only 1.6% of the population aged 80 years and over in 2006 was non-White and this will increase little, to 3.6%, by 2030 [31]. A strength of the Newcastle 85+ study is the diversity of types of data collected (self-reports, proxy reports, clinical measurement) and sources of data (structured interviews, clinical records) that allow comparisons between estimates that use different methods of data collection and sources of data. Appropriate resources and considerable effort were also used to ensure that high quality data were captured [17-19].

Our estimates of the levels of ability to undertake ADLs and interval-need dependency in this age group are consistent with the trends observed in other studies [2,4,5,7,9-11]. Nowadays, older people in at least some countries, are more independent than in previous generations [25,32,33], reflecting the shifting of the burden of chronic illness and disability to later chronological ages. This suggests a rather more optimistic future for people turning 85 and challenges the idea that increasing longevity and life expectancy of successive generations of older people should always be treated with alarm. However, evidence for declining levels of disability is mixed, a comparison of the trends in the prevalence of severe disability in older people in twelve OECD countries concluded that only five countries (Denmark, Finland, Italy, the Netherlands, United States) showed clear evidence of a decline in disability whilst three (Belgium, Japan, Sweden) showed clear increases, two (Australia, Canada) stability and a further two (France and the UK) differing trends depending on the data source [34].

Despite the optimistic scenarios that shift the impact of disability and dependency to later chronological ages, the projected increase in the demand for health and social care services remains a considerable concern for politicians and policy makers and society as a whole, particularly in light of the projected decline in availability of formal and informal carers. It will also have significant implications for the skills training of both qualified and unqualified personnel particularly in relation to the care of people with cognitive impairment. The planning of health and social care services is a major challenge, since forecasting future demand is an inexact science. Future demand is influenced not only by demographic change and the capability and dependency of the older population. The nature of the health and social care systems, the changing structure of social support networks, technological innovations and policy, reflecting the changing attitudes to supporting older people and the use of scarce societal resources, will also influence projections. For this reason we have made a number of gross assumptions in estimating the impact of future levels of capability and dependency. Our assumption of constant prevalence of interval-need dependency is conservative for the UK, and indeed more generally [34]. We accept that our projections are relatively imprecise. However the challenge of population ageing is of a sufficient magnitude to justify attempting these projections.

## Conclusions

The significance of this study is that it highlights the increasing capability of the oldest old but also provides policy makers with robust data and estimates of need about the increasing numbers of very old people around the age to which more than half the population nowadays are expected to survive. Even without the impact of global economic recession on levels of public expenditure the current ways of delivering long-term care are probably unsustainable given the future decline in the number of working-age adults available for employment in the long-term care sector [35]. Innovative solutions to this challenge, involving families and community support networks that utilise the increasing pool of active and capable retired people will be needed to maintain levels of independence among the oldest old and provide appropriate long-term support for those who require 24-hour care or at least regular daily care.

## List of abbreviations

ADL: Activities of Daily Living; K-W: Kruskall-Wallis; GARS: Groningen Activity Restriction Scale; M-W: Mann-Whitney; NHS: National Health Service; OR: Odds ratio; SMMSE: Standardised Mini-Mental State Examination; TUG: Timed up and go test; UK: United Kingdom

## Competing interests

The authors declare that they have no competing interests.

## Authors' contributions

CJ participated in the study design, analysis and interpretation, the conception and writing of the paper; JC participated in the study design and its execution, data collection, data preparation, analysis and interpretation, and the conception and critique of the paper; KD participated in the study design and its execution, data collection, data preparation and critique of the paper; AK participated in the analysis and interpretation, and critique of the paper; LAR participated in recruitment, analysis and critique of the paper; MPE participated in the study design, analysis and critique of the paper; TvZ participated in the study design and critique of the paper; CM-R participated in the study set up and critique of the paper; OFWJ participated in the study design, analysis and critique of the paper; TBLK conceived the study, secured funding, and oversaw all aspects as lead investigator. He participated in the study design and its execution, analysis and critique of the paper; JB participated in the study design, analysis and interpretation, the conception and writing of the paper. CJ and JB are the guarantors. All authors saw and approved the final manuscript, had full access to all of the data (including statistical reports and tables) in the study, and can take responsibility for the integrity of the data, data analysis and its interpretation. All authors read and approved the final draft.

## Pre-publication history

The pre-publication history for this paper can be accessed here:

http://www.biomedcentral.com/1471-2318/11/21/prepub
